# Stigma among UK family carers of people living with dementia

**DOI:** 10.1192/bjo.2022.585

**Published:** 2022-10-07

**Authors:** Jem Bhatt, Katrina Scior, Charlotte R. Stoner, Esme Moniz-Cook, Georgina Charlesworth

**Affiliations:** Research Department of Clinical, Educational and Health Psychology, University College London, UK; Centre for Chronic Illness and Ageing, School of Human Sciences, University of Greenwich, UK; Faculty of Health Sciences, University of Hull, UK; Research Department of Clinical, Educational and Health Psychology, University College London, UK; and Research and Development Department, North East London Foundation Trust, UK

**Keywords:** Stigma and discrimination, carers, dementia, social functioning, attitudes

## Abstract

**Background:**

Models of caregiving seldom include the role of stigma when understanding the experiences of carers of people living with dementia.

**Aims:**

To investigate the validity of the Family Stigma Instrument (FAMSI), and use it to explore the extent to which experiences of stigma are endorsed in family carers of people living with dementia.

**Method:**

The FAMSI was tested with 70 carers of people living with dementia. They also completed a measure of self-esteem.

**Results:**

The FAMSI demonstrated some good preliminary psychometric properties. Carers endorsed stigma by association more so than affiliate stigma constructs, suggesting that carers were aware that others viewed or treated them in a stigmatising fashion but did not endorse internalised consequences of this as much (e.g. behavioural or affective affiliate stigma).

**Conclusions:**

The FAMSI offers new avenues for understanding the contribution of stigma to caregiver burden in dementia. It also captures the positive aspects of caregiving, which may mitigate internalised stigma in family carers, and has good potential for evaluating stigma-neutralising interventions in dementia care.

Carer burden and its predictors have been widely studied in dementia.^1–3^ Studies on the multidimensional measurement of carer burden often fail to consider the contribution of stigma to burden, or the positive aspects of caring that may act to protect family carers.^4^ Stigma directed at family carers of a stigmatised individual is construed as ‘stigma by association’ or ‘courtesy stigma’.^5^ When stigma by association becomes internalised, termed ‘affiliate stigma’, it can have negative affective, behavioural and cognitive consequences, such as unhappiness, withdrawal and sense of inferiority.^6^ Important processes that contribute to or mitigate carer burden, such as stigma by association and positive aspects of caregiving, are absent from instruments used to examine affiliate stigma in carers of people living with dementia.^2,7^ One instrument that does incorporate these processes is the Family Stigma Instrument (FAMSI), devised for use with family carers of people with intellectual and developmental disabilities (IDDs).^8^ The FAMSI is a conceptually driven instrument grounded in both stigma theory and positive psychology approaches to caregiving. As such, it potentially has added utility for understanding the fine balance between positive and negative consequences of dementia for caregiver burden. Our aim was to investigate the validity of the FAMSI in dementia care, and the extent to which experiences of stigma are endorsed in a UK population of family carers of people living with dementia.

## Method

### Setting and participants

Participants were a convenience sample in South-East England, UK. They were recruited via the Join Dementia Research database (https://www.joindementiaresearch.nihr.ac.uk/) and through social media, community advertising or researcher outreach activities where the study was presented to carers’ groups (e.g. Alzheimer's Society groups). Participants were included if they were aged ≥18 years; were a family carer for someone with a primary progressive dementia; and were able to understand, read and write the English language. Participants were excluded if they were a former carer; had a late-stage chronic, terminal medical condition or a significant sensory impairment that would preclude completion of the study measures; or lacked the capacity to consent. Sample size was calculated based on guidance^9^ where seven multiplied by the number of FAMSI items gave the target sample size (e.g. 7 × 26 = 182 participants).

### Data collection

The authors assert that all procedures contributing to this work comply with the ethical standards of the relevant national and institutional committees on human experimentation and with the Helsinki Declaration of 1975, as revised in 2008. All procedures involving human patients were approved by University College London Research Ethics Committee (approval number 11501_002).

Data were collected through face-to-face interviews and online questionnaires, according to participant preference and geographical locality. Potential participants were given a study information sheet and at least 24 h to consider participating, before taking part either face to face with a researcher or independently online. The corresponding author or one of two Master's students carried out face-to-face data collection. Qualtrics version 19 (Qualtrics, Provo, UT, USA, https://www.qualtrics.com) was used for online data collection, with participants accessing the participant information sheet, screening questions, consent form and study measures through a survey link. All participants were asked to complete a retest, for which they completed the measures again after a 2-week period (time point 2) in the same format in which they had completed them initially (time point 1). This retest was to assess the stability of the measure over a 2-week period, which is sufficient time to balance between recollection bias and unwanted change.^10^

### Measures

#### FAMSI

The FAMSI^8^ is a 26-item instrument with two stigma-focused subscales of stigma by association (eight items) and affiliate stigma (affective, behavioural and ‘perceived’ domains with four items each), and a third subscale concerned with positive aspects of caring (six items). Items relating to stigma by association began with the phrase, ‘some people might’, e.g. ‘ … feel embarrassed about associating with them’. Items on the affiliate stigma subscale began with the phrases, ‘I feel … ’, ‘I avoid …. ’ or ‘I am … ’ for the affective, behavioural and perceived domains, respectively. For example, ‘I feel embarrassed about them’ (affective), ‘I avoid introducing my friends to them’ (behavioural), or ‘I am excluded from activities when other people find out about their dementia’ (perceived). The affiliate stigma questions were framed such that ‘them’ or ‘their’ referred to the person living with dementia. The subscale pertaining to perceived affiliate stigma, which Mitter et al^8^ originally labelled ‘cognitive’, was changed to ‘perceived’ as items for this domain reflect family carers’ perceptions of how others treat them. Positive aspects of caregiving items began with the phrase ‘Caring for my family member .… ’, e.g. ‘ … has enabled me to develop a more positive attitude toward life’. Response options for all items were on a five-point scale from ‘strongly disagree’ (1) to ‘strongly agree’ (5), with a midpoint of ‘neither agree nor disagree’ (3). FAMSI instructions and items were adapted to be relevant for carers of people living with dementia by replacing the term ‘intellectual and developmental disability’ with dementia. The original validation of the FAMSI with family carers of people with IDDs demonstrated adequate to good internal consistency for all subscales (Cronbach's alpha range 0.77–0.91). However, only the positive aspects of caregiving and affective affiliate subscales were stable over time (intraclass correlation coefficient (ICC) >0.7).

#### Rosenberg Self-Esteem Scale

The Rosenberg Self-Esteem Scale (RSES)^11^ was used to measure self-esteem of carers. Previous literature has documented an inverse relationship between self-esteem and stigma in dementia, HIV/AIDs and cancer.^12–14^ Therefore, it was hypothesised that stigma by association and affiliate stigma would be negatively correlated with self-esteem, whereas the positive aspects of caregiving subscale would be positively correlated.

#### Demographics questionnaire

The following demographic information was collected through carer report: gender, age, kin relationship to person living with dementia, ethnicity, cohabitation status, employment status and whether English is their first language. In addition, participants were asked the age and dementia subtype of the care recipient, and time since diagnosis.

### Data analysis

Data distributions of the FAMSI (floor/ceiling effects, normality), internal consistency (Cronbach's alpha cut-off value ≥0.70, indicating adequate internal consistency), test–retest reliability (ICC cut-off value ≥0.70, indicating adequate test–retest reliability) and convergent validity with the RSES were analysed in accordance with psychometric guidelines,^9^ using SPSS version 26 for Windows. It was hypothesised that stigma by association and affiliate stigma would be negatively correlated with RSES score, and positive aspects of caregiving would be positively correlated with RSES score. Percentages for each response option were compared for all FAMSI items, to explore which FAMSI domains were endorsed over others. Relationships between domains were explored through Pearson's correlations, as per previous analysis.^8^

## Results

### Sample characteristics

Seventy carers of people living with dementia met the eligibility criteria and provided informed consent. Four participants took part face to face and 66 participants took part online (see Table 1). An additional three online participants dropped out during screening and one online participant was excluded because of a high level of incomplete data (>30% of items unanswered). Across all measures, levels of missing data were low (<10%), indicative of high completion rates and acceptability and suitability of the measures in this population. A Little's missing completely at random test was non-significant for each measure (*p* = 0.623), indicating data were missing completely at random. Therefore mean imputation at an item level was performed to deal with the low levels of missing data.^15,16^

**Table 1:** Participant characteristics and demographics (*N* = 70)

		Mean (s.d.) or *n*
Carers		
Age, years		60.00 (13.19); range 27−87
Gender (male/female/not disclosed)		16/53/1
Ethnicity	White	65
	Black/African/Caribbean	1
	Mixed multiple	1
	Other ethnic group	2
	Not disclosed	1
Relationship to person living with dementia	Spouse/partner	24
	Child/child-in-law	38
	Other	7
	Not disclosed	1
Employment status	Employed	32
	Retired	35
	Other	2
	Not disclosed	1
English as first language	Yes	68
	No	1
	Not disclosed	1
Persons living with dementia		
Age, years/range		80.61 (7.76); range 63−100
Months since diagnosis		69.04 (47.61)
Type of dementia	Alzheimer's disease	26
	Vascular dementia	13
	Frontotemporal dementia (behavioural variant)	8
	Lewy body	2
	Mixed	17
	Not disclosed/unknown	4

Participants were on average 60 years of age and predominantly White (92%), female (76%) and caring for their own or a spouse's parent (54%). The person they cared for was most commonly living with Alzheimer's disease (37%) or dementia of mixed aetiology (24%).

### Assessment of psychometric properties of FAMSI domains

FAMSI subscales were normally distributed, with the exception of the stigma by association subscale where data were moderately negatively skewed (−1.255), although this was below the absolute skew limit of −2 (see Table 2). Analysis indicated some potential floor effects of the affective affiliate stigma and behavioural affiliate stigma subscales, with 32.9% and 30% of the sample achieving the lowest score for these measures, respectively. All FAMSI scales had adequate test–retest reliability and internal consistency.^9^ No significant correlations between the FAMSI scales and RSES were observed, indicating a lack of convergent validity.

**Table 2:** Reliability and validity statistics of the Family Stigma Instrument

FAMSI domains	Distribution of data			Floor and ceiling				Reliability				Convergent validity
	Mean (s.d.)	Skewness	Kurtosis	Minimum	Maximum	Lowest score (%)	Highest score (%)	Internal consistency (*α*)	Test–retest			Pearson's *r*
									ICC	Lower	Upper	
Stigma by association	27.37 (6.96)	−1.255	1.630	8	38	5.7	0	0.917	0.822	0.699	0.894	0.098^a^
Positive aspects of caregiving	19.26 (4.39)	−0.046	0.160	8	30	0	2.9	0.720	0.832	0.715	0.900	0.039
Affiliate stigma (total)	25.76 (7.51)	0.188	−0.223	12	44	5.7	0	0.858	0.728	0.544	0.840	−0.120
Affective	6.93 (2.93)	0.694	−0.640	4	14	32.9	0	0.857	0.749	0.577	0.851	−0.183
Perceived	12.09 (4.23)	−0.429	−0.625	4	20	7.1	2.9	0.875	0.818	0.696	0.893	0.023
Behavioural	6.74 (2.56)	0.794	0.073	4	14	30.0	0	0.759	0.746	0.570	0.850	−0.181

FAMSI, Family Stigma Instrument; ICC, intraclass correlation coefficient.

a. Use of non-parametric test.

### Endorsement of FAMSI domains

Response options endorsed by carers for each item of the FAMSI are presented in Table 3. Overall, carers agreed with statements in the stigma by association scale. Overall, the participants rejected the affective and behavioural domains of affiliate stigma, suggesting that they were able to resist the potential negative emotional impact of stigma and did not change their own behaviour when in social situations. In contrast, they tended to endorse items in the perceived domain, indicating that they perceived a change in others’ behaviour toward them as a result of their relative's diagnosis of dementia. Although there was some agreement that caring for someone with dementia allowed them to form friendships with others in a similar situation, no other positive aspects of caring were either strongly endorsed or rejected by the sample as a whole.

**Table 3:** Endorsement ratings of the Family Stigma Instrument domains

				Endorsement of each response option, *n* (%)				
FAMSI domain		Item wording		Strongly disagree	Somewhat disagree	Neither disagree/agree	Somewhat agree	Strongly agree
Stigma by association	Some people might …	1	feel embarrassed about associating with them	6 (8.6)	13 (18.6)	6 (8.6)	37 (52.9)	8 (11.4)
		2	feel uncomfortable about going to their house	4 (5.7)	5 (7.1)	6 (8.6)	41 (58.6)	14 (20.0)
		3	treat them more negatively	4 (5.7)	6 (8.6)	10 (14.3)	40 (57.1)	10 (14.3)
		4	think that the family has done something wrong because of them	14 (20.0)	27 (38.6)	20 (28.6)	5 (7.1)	4 (5.7)
		5	behave negatively toward them when they are with the person living with dementia in public	7 (10.0)	11 (15.7)	16 (22.9)	28 (40.0)	8 (11.4)
		6	avoid making friends with them	5 (7.1)	6 (8.6)	11 (15.7)	40 (57.1)	8 (11.4)
		7	not want to hear about any of their problems	5 (7.1)	8 (11.4)	7 (10.0)	40 (57.1)	10 (14.3)
		8	not invite the family to social events	5 (7.1)	7 (10.0)	10 (14.3)	32 (45.7)	16 (22.9)
Positive aspects of caregiving	Caring for my family member living with dementia has …	9	enabled me to develop a more positive attitude toward life	5 (7.1)	24 (34.3)	16 (22.9)	17 (24.3)	8 (11.4)
		10	made me feel needed	0 (0)	11 (15.7)	21 (30.0)	25 (35.7)	13 (18.6)
		11	strengthened my spirituality and faith	15 (21.4)	17 (24.3)	23 (32.9)	8 (11.4)	7 (10.0)
		12	allowed me to form friendships with others in a similar situation	6 (8.6)	10 (14.3)	9 (12.9)	34 (48.6)	11 (15.7)
		13	made me feel that I make a positive contribution to society	4 (5.7)	12 (17.1)	21 (30.0)	24 (34.3)	9 (12.9)
		14	strengthened some of my relationships with family/friends	4 (5.7)	18 (25.7)	14 (20.0)	24 (34.3)	10 (14.3)
Affective affiliate stigma	I feel …	15	embarrassed about them (my family member living with dementia)	30 (42.9)	21 (30.0)	12 (17.1)	7 (10.0)	0 (0)
		16	distressed about being associated with them	37 (52.9)	25 (35.7)	6 (8.6)	2 (2.9)	0 (0)
		17	guilty about having them in the family	48 (68.6)	17 (24.3)	2 (5.7)	1 (1.4)	0 (0)
		18	uncomfortable when I have friends about because of them	28 (40.0)	24 (34.3)	10 (14.3)	8 (11.4)	0 (0)
Perceived affiliate stigma	I am …	19	treated differently by some people when I am with them	12 (17.1)	9 (12.9)	14 (20.0)	29 (41.4)	6 (8.6)
		20	excluded from activities when other people find out about their dementia	14 (20.0)	20 (28.6)	13 (18.6)	18 (25.7)	5 (7.1)
		21	aware of how some people look at me when I am out with them	11 (15.7)	14 (20.0)	14 (20.0)	26 (37.1)	5 (7.1)
		22	treated differently by some people because of them	11 (15.7)	6 (8.6)	13 (18.6)	34 (48.6)	6 (8.6)
Behavioural affiliate stigma	I avoid …	23	introducing my friends to them	28 (40.0)	23 (32.9)	8 (11.4)	11 (15.7)	0 (0)
		24	telling people that I am related to them	45 (64.3)	22 (31.4)	3 (4.3)	−	0 (0)
		25	making new friends because of them	31 (44.3)	25 (35.7)	8 (11.4)	6 (8.6)	0 (0)
		26	being seen with them	43 (61.4)	22 (31.4)	4 (5.7)	1 (1.4)	0 (0)

FAMSI, Family Stigma Instrument.

### Relationships between FAMSI domains

A moderate significant correlation between stigma by association and both affiliate stigma (*r* = 0.489, *P* < 0.001) and perceived affiliate stigma (*r* = 0.624, *P* < 0.001) was observed, indicating that carers who endorsed stigma by association items were also more likely to experience affiliate stigma. Perceived affiliate stigma was positively correlated with affective (*r* = 0.272, *P* < 0.05) and behavioural family stigma (*r* = 0.300, *P* < 0.05), and behavioural and affective family stigma were positively correlated with each other (*r* = 0.670, *P* < 0.001).

## Discussion

Stigma in dementia caregiving is an emerging area of research.^6,7^ This study is, to our knowledge, the first quantitative exploration of stigma by association, affiliate stigma and positive aspects of caregiving with family carers of people living with dementia in the UK. This study adds to the notion of the ‘double effect’ of dementia on experiences of stigma, where people living with dementia experience stigma^14^ and the majority of their carers also experience stigma. Our results show that carers endorsed stigma by association, suggesting they were aware that others viewed or treated them in a stigmatising fashion; however, the lack of endorsement of affiliate stigma constructs (e.g. behavioural and affective) suggests that carers may not have experienced the internalised consequences of stigma as strongly, and reasons for this are discussed below. Alongside stigma, many carers reported positive experiences within caregiving. This suggests a complex relationship between stigma experiences and caregiving, whereby carers experience and are aware of both positive and negative aspects of their role, and therefore positive aspects of caregiving may mediate or buffer against stigma. This confirms other work noting the coexistence of positive and negative aspects in caregiving.^17^

### Polarising versus balancing the experiences of carers

Three instruments are available to quantify affiliate stigma, namely the Family Stigma in Alzheimer's Disease Scale,^2^ the Affiliate Stigma Scale^7^ (developed for caregivers of people with mental health challenges) and the FAMSI (developed for people with IDDs).^8^ Limitations of the first two instruments include a focus on negative experiences^2^ or neglect of stigma by association.^7^ To further our understanding of carer burden, stigma and the balance of negative and positive experiences in families, we chose to examine the psychometric properties of the FAMSI as it is the only instrument that encompasses stigma by association and affiliate stigma, as well as positive aspects of caregiving. The present psychometric evaluation of the FAMSI in dementia care suggests good validity for use with families. Analysis indicates that it has acceptable internal consistency and content validity, with the latter evidenced by significant correlations between total FAMSI scores and its subscales.

Our validation of the FAMSI measure in dementia caregiving offers new avenues for further exploration of the wide-ranging experiences of stigma, burden and emerging positive psychology approaches.^18^ For example, future research with the FAMSI could investigate how positive frames in caregiving may counteract stigma by association, stigmatised beliefs or feelings of shame that contribute to increased burden and reports of behavioural and psychological symptoms in dementia.^19^ Although stigma resistance has been explored across mental health conditions, there are no such explorations in the field of dementia caregiving. The FAMSI is an appropriate tool for further examination of this and the relationship between positive aspects of caregiving and stigma by association in dementia.

The theoretical model of sense of competence, which incorporates burden and positive aspects of caregiving, has been prevalent in the dementia caregiving literature for some time. However, despite significant experience of stigma by association, as documented here, this concept has yet to be included in formal models of caregiving. As such, this research represents the first step in quantitatively documenting stigma by association in caregivers and its relationship with positive aspects of caregiving.

### Relevance of stigma to carers of people living with dementia

This study provides scope for further use of the FAMSI in conceptual work, as well as being mindful in practice that stigma is often felt by those living with dementia and those caring for them.^20^ Experiences of perceived affiliate stigma were more heavily endorsed compared with affective or behavioural affiliate stigma. Carers were almost eight times more likely to report perceived affiliate stigma compared with other types of affiliate stigma. Of note is our adaption of the label, but not the item descriptions, of the affiliate stigma scale, i.e. ‘cognitive’ altered to the ‘perceived’ domain. This is in line with the dementia literature where perception is seen as a particular aspect of cognition relating to a person's view or understanding of their experience.^19^ In this case, items of this domain reflected the perceptions of carers and how they experienced the behaviours of others as stigmatising (e.g. I am treated different by some people when I am with them), rather than reflecting the cognitions of carers that may be internalised as a result of being stigmatised.

Similar to previous findings in the IDD field,^8^ more than half of the carers perceived the role of being a family carer as stigmatising. Many carers reported positive aspects of their caring roles. It is possible, for example, that if carers frame their role positively, they may be more likely to be able to resist shame and other negative feelings that have been associated within their reports of ‘behavioural and psychological symptoms in dementia’^19,21^ during interactions with their relative with a dementia or within their caring role. Thus, in their journey of dementia care, they may become less vulnerable to these aspects of affiliate stigma. This may also explain why, as compared with other domains, there was strong endorsement of perceived affiliate stigma, since positive framing of a caring role or stigma resistance^22^ may be less likely to affect experiences of affiliate stigma. These findings suggest that affective and behavioural affiliate stigma reflect the carer's own feelings and responses, whereas perceived affiliate stigma is more reliant on external factors such as the carer's perception of the behaviour of others toward them within their social environment.

### Limitations

Our study has several limitations that should be considered for future research. First, despite efforts to recruit a large and diverse sample, we did not meet our recruitment target, and the majority of participants were White and lacked ethnic diversity. Given knowledge of stigma experiences in family carers and ethnicity in the UK,^23^ it will be important to examine the validity of the FAMSI with distinct groups of family carers in the UK. Also the validity of the FAMSI cannot be generalised to family stigma and dementia care in other countries and cultures.^24^

Second, the FAMSI was originally developed to understand the experiences of family carers of people with IDDs and adapted for the dementia field, and not developed as a scale unique to the experiences of family carers of people living with dementia. It is conceivable that some of their specific concerns in relation to stigma may have been missed as a result.

Finally, self-esteem scores were not significantly correlated with stigma by association as measured by the FAMSI. This may have been because of the small sample size, but since the link between the concepts of self-esteem and stigma is based on research on the individual with a stigmatising condition, it is also possible that self-esteem is not theoretically related to stigma by association.^25^ For instance, carers are fundamental to ensuring that the needs of the person they care for are met on a daily basis. This has strong connotations with a sense of purpose and it is possible that, having been primed with the FAMSI, carers completed the RSES foregrounding a salient carer identity rather than other personal identities. Future studies could explore measurement of other concepts related to stigma by association, such as its role in the management of unmet need in people with dementia and behavioural and psychological symptoms in dementia. Methodology could also involve randomisation of the order in which measures are presented to participants.

### Implications for clinical practice and future research

This research has laid the foundation for use of the FAMSI in dementia care, offering an instrument to further our understanding of stigma felt by family caregivers. Prevalent models of burden neglect stigma and its potential consequences for family caregiving. Theoretical models should be further developed to investigate stigma and its relationship with distress and burden, as well as positive psychological care, in family care settings.

Our study notes that the majority of carers experience stigma, but there are no widely implemented interventions targeting this in dementia care. This study has implications for health and social care practice, where the experience of carers should be acknowledged in formal care pathways and relevant support offered. For example, psychosocial factors have a known effect on behavioural and psychological symptoms in dementia (e.g. neuropsychiatric disturbances, distress in families),^26^ where the roles of stigmatised beliefs and shame have also been implicated.^19^ Using the FAMSI in formal assessments provides scope for understanding the hidden needs of families. Tailored stigma-neutralising interventions for family carers vulnerable to the effects of stigma need to be developed, and their impact on ongoing burden evaluated within longitudinal studies.

### Future research

Further research in this field should aim to identify the impact of positive aspects of caregiving, stigma by association and affiliate stigma on carers, to determine vulnerabilities that may contribute to caregiver burden. Studies could examine hypotheses predicting that greater perceptions of positive aspects of caregiving have health and social benefits for carers. Recent literature has found that positive aspects of caregiving have been associated with benefits for carers, but not people living with dementia.^21^ However, past literature has not fully incorporated stigma into both parts of the dyad or examined its relative contribution to quality of life for people living with dementia and their families. In addition, future research should aim to test the factor structure of the FAMSI.

## Data Availability

The data that support the findings of this study are available from the corresponding author, J.B., upon reasonable request.
